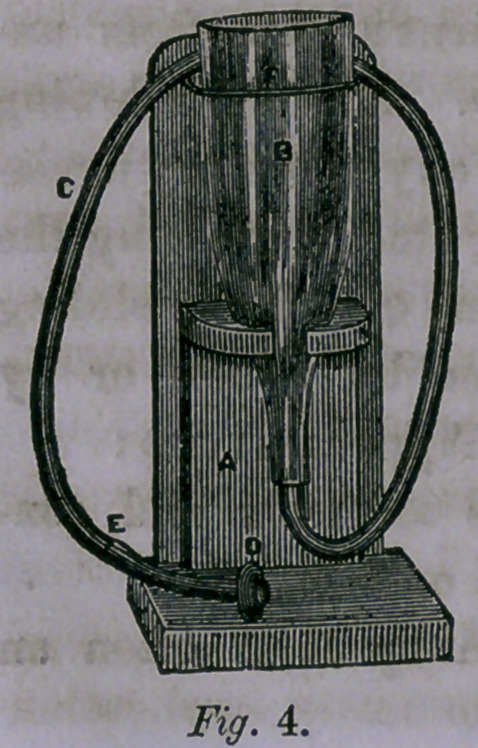# Chicago Medical Society

**Published:** 1866-08

**Authors:** 


### PROCEEDINGS OF MEDICAL SOCIETIES.

### CHICAGO MEDICAL SOCIETY.

During the past month the weekly meetings of the society have given place to a monthly session, according to the time-honored usages appropriate to the heated term of summer. Our report must, therefore, be rather meagre.

### RETENTION OF THE MENSES.

Dr. Orrin Smith reported the case of a married woman who, for two years, had retained her catamenia. The molimen occurred regularly every month, and the abdomen was much enlarged. The speculum showed that the os uteri was closed by adhesion of the lips. The patient had been under treatment for ulceration of the os and cervix previous to the commencement of retention. Dr. S. proceeded to relieve the patient by forcing a bougie through the cicatrix. The uterine cavity was entered without difficulty, and several pints of dark, grumous fluid were withdrawn. With appropriate treatment the woman recovered, and is now menstruating in a normal manner.

### NEW APPARATUS.

Dr. Holmes exhibited and explained several varieties of spray producing instruments, or, as they are sometimes called, atomizers or pulverizers; also the instrument for producing local anæsthesia by freezing, and the apparatus for irrigating the nasal cavities with medicated fluids.

The essential portion of nearly all instruments for producing spray consists in a simple arrangement of two small tubes, (Bergson's Atomizing Tubes,) each terminating at one end in a very minute opening. These tubes are united at right angles with each other, the minute openings being in close juxtaposition. If one of these tubes be dipped in any thin fluid, and a current of air or steam (fig. 1) forced through the other, the fluid will be drawn up the first tube and carried in a jet of fine spray by the air or steam passing from the other tube.

Any soluble medicament can thus be carried in the spray either by inhalation or by the *direct* force of the lungs, fauces or nasal cavities.

This manner of applying astringents and other medicinal agents to the mucous membranes of the air passages has been found by experience not only very convenient but attended with most beneficial results. In cases requiring the use of simple vapor, or that impregnated with lime, as for croup and diphtheria, some form of spray producer will be found convenient, since it can be readily put in operation under a suitable covering thrown over the patient's bed. The instruments may also be used for the diffusion of perfumes and disinfectants in the sick chamber.

One of the most simple and convenient forms of apparatus for inhalation is, perhaps, that (fig. 2) devised by Dr. W. K. Oliver, of Boston, who has devoted much attention to the study and treatment of diseases of the air passages. It consists of a kind of double-mouthed bottle, which contains the spray-producing tubes, the medicated fluid being placed at the bottom of the bottle. The spray is produced by forcing air through the tube by means of pressure of the hand upon a hollow india-rubber ball, as in a well known form of syringe.

The spray is formed within the bottle; on applying the mouth to one of the openings, the vapor is inhaled as rapidly as produced.

A very small form of atomizer may be used for producing a kind of douche for the eye, or, in fact, for any of the purposes just mentioned.

The freezer (fig. 3) consists of metallic tubes, constructed upon the same principles as the tubes, already described. The most suitable fluids from which the spray may be produced have been found to be either sulphuric æther or rhigolene. This last is a hydro-carbon, with a specific gravity of .625, boils at 70° Fahrenheit, and is the lightest known fluid. It is nearly identical with kerosolene, an efficient anæsthetic, as some four members can testify by actual personal experience, some years since.

The spray of æther or rhigolene from the point of this instrument, held near any portion of the body, will speedily produce congelation and anæsthesia, as may be seen by the blanched appearance and by the insensibility on incising the part. This has been shown to be applicable in many of the minor but painful operations. No inflammatory action has been observed to take place in the tissues subjected to the influence of these agents.

[Through the kindness of Messrs. Codman & Shurtleff, of Boston, and a friend, who recently visited that city, we are able to present our readers with the following excellent diagrams and descriptions of the instruments above mentioned:]

*Fig.* 1.

*Figure* 1, *Steam Atomizing Apparatus.*—This consists of a Boiler, in which steam is generated by flame of lamp J. J, Lamp, provided with tube for graduating flame for much or little heat. K, Safety valve, graduating to high or low pressure. By unscrewing the valve tube from its position, the boiler may be supplied with water without disturbing the atomizing tubes. L, Milled button or top. Between this and a suitable projection or shelf within the neck of the boiler, is secured the packing of rubber through which the atomizing tube passes — air and steam tight. M, Wood ferrule to protect the hand from heat in removing the boiler and tubes for the purpose of changing the medicament. N, The atomizing tubes. O, Cup in which the medicament is placed. P, Shield for protecting the patient's face from unpleasant contact with the medicated vapors. Q, Joint allowing the shield to be moved to, and retained at, any necessary deviation from a horizontal position. R, Sliding staff regulating the height of the shield. By means of the joint Q and the sliding staff, the shield may be adjusted for use by adults or by children. Its advantages over all the Air Apparatus are:

1st. That being self-acting, it produces an even and continuous flow of spray without inconvenience or labor.

2d. The warmth of the spray produced by it is often an advantage.

*Fig.* 2.

*Fig.* 2 represents Oliver's *Atomizer and Inhaler.* A, Elastic Bulb with Valves, serving as a bellows to produce the spray within the jar. B, The Bergson Atomizing Tubes, the upright arm being formed in part by a rubber tube, which dips into the medicament placed in the bottom of the jar. C, Opening for the admission of air. The advantages of Dr. Oliver's instrument over all other hand instruments, consists in this:

1st. That the receptacle for the medicament and the shield for the protection of the face are united in one piece.

2d. That none but the finest of the particles of spray are inhaled.

3d. That the Bergson tubes being within the jar, are protected from injury.

*Fig.* 3.

*Fig.* 3, *Freezing Apparatus for producing Local Anœsthesia.*—This form of apparatus is all that is required for producing Local Anæsthesia by freezing with Ether, as employed by Dr. Richardson, of London, or with Rhigolene, as described by Dr. H. J. Bigelow, of Boston, in the *Boston Medical and Surgical Journal* of April 19th, 1866.

*Fig.* 4.

*Fig.* 4.—A form of *Nasal Douche* for the treatment of diseases of the nasal passages, as described by Prof. Thudichum. A, Black Walnut Stand. B, Conical Reservoir. C, Leading Tube. D, Nozzle. E, Joint. F, Ring, hinged to stand to support the Reservoir.

In using this kind of Douche, the Reservoir is placed higher than the head, and the rubber tube is grasped near the nozzle, between the thumb and finger, so as to control the current. The nozzle is then depressed enough to allow a little of the liquid to escape, thereby expelling air from the tube. It is then pressed gently into the nostril, and the grasp slightly relaxed, when the current will enter and fill the whole cavity of the nose and escape by the opposite nostril, the head at this time being thrown slightly forward over a basin, and the mouth kept open.

These instruments, with pure rhigolene, s. gravity, .625, as also pamphlets describing their use, with papers by distinguished medical men on this mode of treating certain diseases, may be obtained on application to Messrs. Codman & Shurtleff, 13 & 15 Tremont street, Boston, or to their agents, Messrs. Bliss & Sharp, Druggists and Apothecaries, 144 Lake street, Chicago.

## Figures and Tables

**Fig. 1. f1:**
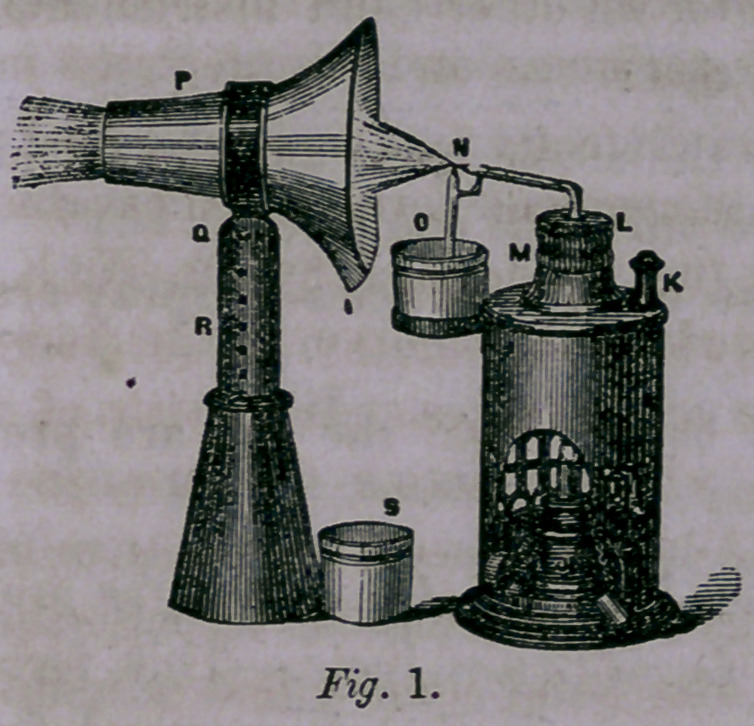


**Fig. 2. f2:**
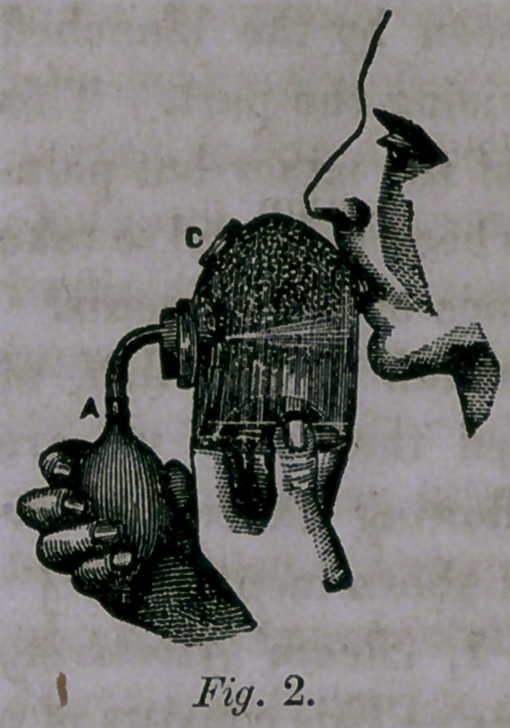


**Fig. 3. f3:**
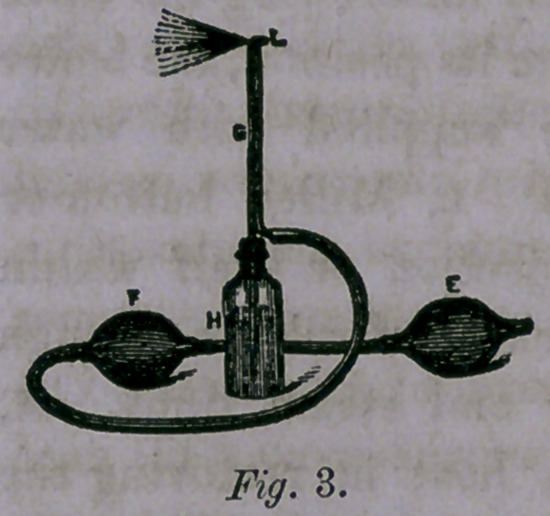


**Fig. 4. f4:**